# Supplementary material for: Development and Validation of the Artificial Intelligence in Mental Health Scale: Application for AI Mental Health Chatbots
Source: Healthcare (Basel). 2025 Dec 12;13(24):3269. doi: 10.3390/healthcare13243269 (PMC12732789; doi:10.3390/healthcare13243269)
Supplement: Supplementary file 1 [file healthcare-13-03269-s001.zip › Supplementary Table S1.pdf]

Supplementary Table S1. The 14 items that were produced after the assessment of the content and face validity of the Artificial Intelligence in Mental Health Scale.

Introductory note

Artificial Intelligence (AI) chatbots are emerging as a tool for mental health support, offering accessible and convenient assistance to individuals. These AI chatbots simulate human conversation, and they can learn from interactions, improve over time, and handle a wide range of queries. For instance, AI chatbots can provide emotional support, coping mechanisms, and even guidance on managing symptoms. AI chatbots cannot replace traditional therapy, but they can be a helpful resource by offering support, guidance, and a sense of connection. There are free chatbots, while more sophisticated models with more features require a low-cost subscription.

We are interested in your attitudes towards the use of AI chatbots for mental health support. Please complete the following scale, indicating your response to each item. There is no right or wrong answers. We are interested in your personal views.

**Artificial intelligence chatbots …**
**1. cannot understand people's emotions**
2. can demonstrate sufficient social and cultural sensitivity to support diverse populations
**3. cannot provide correct information on mental health issues**
**4. cannot achieve empathy levels comparable to those of a human therapist**
**5. cannot appropriately respond to mental health emergencies (e.g. suicidal thoughts)**
6. can demonstrate better problem-solving skills compared to a human therapist
7. can expand access to mental health care by reducing geographic barriers
8. can expand access to mental health care by providing continuous access (24/7

	availability)
9.	can expand access to mental health care by reducing financial barriers
10.	can eliminate social stigma concerns since users maintain their privacy
**11.**	**cannot improve people's mood**
12.	can offer up to date information on mental health issues
13.	can empower people by providing them with personalized support
**14.**	**cannot enhance early detection for mental health conditions**

Negative items are in bold.
