# Supplementary material for: Development and Validation of the Artificial Intelligence in Mental Health Scale: Application for AI Mental Health Chatbots
Source: Healthcare (Basel). 2025 Dec 12;13(24):3269. doi: 10.3390/healthcare13243269 (PMC12732789; doi:10.3390/healthcare13243269)
Supplement: Supplementary file 1 [file healthcare-13-03269-s001.zip › Supplementary Table S2.pdf]

Supplementary Table S2. Inter-item correlations between the 14 items that were produced after the assessment of the content and face validity of the Artificial Intelligence in Mental Health Scale (n=428).

**Item**	**2**	**3**	**4**	**5**	**6**	**7**	**8**	**9**	**10**	**11**	**12**	**13**	**14**
**1**	0.58	0.42	0.47	0.32	0.34	0.34	0.37	0.32	0.21	0.28	0.27	0.33	0.34
**2**		0.40	0.31	0.37	0.32	0.45	0.47	0.41	0.28	0.29	0.33	0.35	0.32
**3**			0.39	0.21	0.37	0.36	0.42	0.39	0.27	0.37	0.47	0.35	0.34
**4**				0.34	0.57	0.29	0.25	0.17	0.16	0.22	0.26	0.42	0.33
**5**					0.39	0.38	0.37	0.27	0.21	0.27	0.22	0.40	0.38
**6**						0.34	0.31	0.25	0.25	0.28	0.33	0.43	0.39
**7**							0.75	0.63	0.39	0.31	0.39	0.41	0.43
**8**								0.72	0.44	0.38	0.38	0.46	0.40
**9**									0.47	0.35	0.40	0.36	0.29
**10**										0.27	0.37	0.27	0.29
**11**											0.39	0.45	0.37
**12**												0.46	0.43
**13**													0.55
