# Supplementary material for: Development and Validation of the Artificial Intelligence in Mental Health Scale: Application for AI Mental Health Chatbots
Source: Healthcare (Basel). 2025 Dec 12;13(24):3269. doi: 10.3390/healthcare13243269 (PMC12732789; doi:10.3390/healthcare13243269)
Supplement: Supplementary file 1 [file healthcare-13-03269-s001.zip › Supplementary Table S3.pdf]

Supplementary Table S3. Final version of the Artificial Intelligence in Mental Health Scale.

Introductory note

Artificial Intelligence (AI) chatbots are emerging as a tool for mental health support, offering accessible and convenient assistance to individuals. These AI chatbots simulate human conversation, and they can learn from interactions, improve over time, and handle a wide range of queries. For instance, AI chatbots can provide emotional support, coping mechanisms, and even guidance on managing symptoms. AI chatbots cannot replace traditional therapy, but they can be a helpful resource by offering support, guidance, and a sense of connection. There are free chatbots, while more sophisticated models with more features require a low-cost subscription. We are interested in your attitudes towards the use of AI chatbots for mental health support. Please complete the following scale, indicating your response to each item. There is no right or wrong answers. We are interested in your personal views.

**Artificial intelligence chatbots …**	**Answers**				
	**Strongly disagree**	**Disagree**	**Neither disagree nor agree**	**Agree**	**Strongly agree**
1. cannot achieve empathy levels comparable to those of a human therapist	5	4	3	2	1
2. can demonstrate better problem-solving skills compared to a human therapist	1	2	3	4	5
3. can expand access to mental health care by reducing geographic barriers	1	2	3	4	5
4. can expand access to mental health care by providing continuous access (24/7 availability)	1	2	3	4	5
5. can expand access to mental health care by	1	2	3	4	5

reducing financial barriers

Scoring instructions

Two factors:

- Technical advantages (two items; #1, #2)
- Personal advantages (three items; #3, #4, #5)

Total Score on scale: Adding up the responses to the five items and dividing by 5 gives the total score on the scale. Total score ranges from 1 to 5. Higher scores indicate more positive attitudes towards AI mental health chatbots.

Score on each factor: Adding up the responses to the items in the factor and dividing by the total number of items yields the total score on the factor. Total score on each factor ranges from 1 to 5. For example, in the factor "technical advantages", sum the item responses #1 and #2, and divide by 2. Also, in the factor "personal advantages", sum the item responses #3, #4 and #5, and divide by 3. Higher scores indicate more positive attitudes towards AI mental health chatbots.
