# Supplementary material for: Development and Validation of the Artificial Intelligence in Mental Health Scale: Application for AI Mental Health Chatbots
Source: Healthcare (Basel). 2025 Dec 12;13(24):3269. doi: 10.3390/healthcare13243269 (PMC12732789; doi:10.3390/healthcare13243269)
Supplement: Supplementary file 1 [file healthcare-13-03269-s001.zip › Supplementary Table S4.pdf]

Supplementary Table S4. Corrected item-total correlations, and Cronbach's alpha when a single item was deleted for the five items of the final structure model of the Artificial Intelligence in Mental Health Scale.

**Artificial intelligence chatbots …**	**Corrected item-total correlation**	**Cronbach's alpha if item deleted**
1. cannot achieve empathy levels comparable to those of a human therapist	0.451	0.792
2. can demonstrate better problem-solving skills compared to a human therapist	0.458	0.794
3. can expand access to mental health care by reducing geographic barriers	0.715	0.711
4. can expand access to mental health care by providing continuous access (24/7 availability)	0.734	0.707
5. can expand access to mental health care by reducing financial barriers	0.607	0.750
