# Supplementary material for: Development and Validation of the Artificial Intelligence in Mental Health Scale: Application for AI Mental Health Chatbots
Source: Healthcare (Basel). 2025 Dec 12;13(24):3269. doi: 10.3390/healthcare13243269 (PMC12732789; doi:10.3390/healthcare13243269)
Supplement: Supplementary file 1 [file healthcare-13-03269-s001.zip › Supplementary Table S5.pdf]

Supplementary Table S5. Cohen's kappa for the two-factor five-item model of the Artificial Intelligence in Mental Health Scale.

Artificial intelligence chatbots …	Cohen's kappa	P-value
1. cannot achieve empathy levels comparable to those of a human therapist	0.760	<0.001
2. can demonstrate better problem-solving skills compared to a human therapist	0.785	<0.001
3. can expand access to mental health care by reducing geographic barriers	0.756	<0.001
4. can expand access to mental health care by providing continuous access (24/7 availability)	0.780	<0.001
5. can expand access to mental health care by reducing financial barriers	0.848	<0.001
